# Valorization of Strawberry Tree Berries and Beeswax from Montesinho Natural Park for Cosmetic Industry—A Case Study Formulation

**DOI:** 10.3390/antiox13101152

**Published:** 2024-09-24

**Authors:** Mariana Lamas, Ana Margarida Silva, Manuela M. Moreira, Maria Luz Maia, Valentina F. Domingues, Cristina Delerue-Matos, Maria Helena Amaral, Virgínia Cruz Fernandes, Francisca Rodrigues

**Affiliations:** 1 REQUIMTE/LAQV, ISEP, Polytechnic of Porto, Rua Dr. António Bernardino de Almeida 431, 4249-015 Porto, Portugal; 2 UCIBIO—Applied Molecular Biosciences Unit, MedTech-Laboratory of Pharmaceutical Technology, Faculty of Pharmacy, University of Porto, Rua de Jorge Viterbo Ferreira, 228, 4050-313 Porto, Portugal; 3 Associate Laboratory i4HB—Institute for Health and Bioeconomy, Faculty of Pharmacy, University of Porto, 4050-313 Porto, Portugal

**Keywords:** natural compounds, sustainability, valorization, skin care

## Abstract

Consumers are increasingly concerned about cosmetic ingredients’ origin, looking more than ever to sustainable and greener formulations. The Natural Park of Montesinho, located in Portugal, is characterized by an enormous fauna and flora diversity. Among them, beeswax and strawberry trees (*Arbutus unedo*) have attracted the cosmetic researchers’ interest due to their bioactive compounds’ richness, particularly fatty acids and phenolic compounds. The main goal of this study was to develop an innovative cosmetic product with antioxidant properties composed by both matrices. Briefly, samples were obtained in the Natural Park of Montesinho in October 2022. Beeswax was analysed for lipid profile and contaminants, while extracts were obtained from fruits by ultrasound-assisted extraction (UAE) using water as a solvent. The effect of extraction time (15–90 min) was studied on the total phenolic content (TPC), in vitro antioxidant/antiradical activity, and reactive oxygen species (ROS) scavenging capacity. The beeswax lipid profile presented a high incidence of palmitic, oleic, and linoleic acids. The extract obtained at 60 min presented the highest TPC (30.27 mg GAE/g dw) and antioxidant/antiradical activities (ABTS = 30.36 mg AAE/g dw; DPPH = 43.83 mg TE/g dw; FRAP = 415.61 µmol FSE/g dw). An IC_50_ of 19.78 µg/mL was achieved for the hypochlorous acid, while for superoxide radical and peroxyl radical the IC_50_ were, respectively, 90.51 µg/mL and 0.19 µmol TE/mg dw. The phytochemical profile revealed a high content of gallic acid, and catechin and its derivatives. The hydrophilic cream developed revealed ideal technological parameters, particularly its stability.

## 1. Introduction

Since ancient times, plant- and animal-based ingredients have played a key role in traditional medicine and healing practices, being incorporated into various topical formulations. In the contemporary era, also fueled by consumers’ concerns related to synthetic compounds, a resurgence of natural ingredients has been observed in formulations. Hence, the cosmetic industry has increased its efforts in exploring novel sources of natural ingredients with bioactive properties, such as antioxidant and anti-inflammatory properties [1]. A broad range of natural products with bioactive properties are currently being used within skin care, such as açaí [2] and goji berry extracts [3] for anti-aging purposes, blackberry and raspberry [4], and *Castanea sativa* shell [5], leaf [6], and bur [7] as antioxidant-enriched extracts. These outstanding properties are due to the polyphenolic content of natural compounds. Polyphenols are secondary metabolites that modulate metabolic processes [8] and, based on their chemical composition, are associated with antioxidant, anti-inflammatory, antimicrobial, and anticarcinogenic properties [9]. The polyphenols’ impact varies according to the class, quantity ingested, bioavailability, and origin [8].

Beeswax is a well-known by-product of worker bees, being produced by *Apis melifera* [10]. The European Food Safety Society defines beeswax as “a complex mixture of saturated and unsaturated linear and complex monoesters, hydrocarbons, free fatty acids, free fatty alcohols and other minor exogenous substances” [11]. Various factors can impact the composition of beeswax, such as species, wax age, geographical location, and environmental conditions [12,13,14,15]. Beeswax is not an irritant to the skin and presents low comedogenic potential, as well as anti-inflammatory, antimicrobial, and antioxidant properties that are of interest for the cosmetic industry [15]. In fact, beeswax has been identified in the composition of the first cosmetic cream [16]. In the cosmetic industry, two types of wax are mainly used: yellow (*Cera flava*) and white (*Cera alba*), being commonly employed as base ingredients for different formulas (such as emollient creams, lipsticks, etc.). Furthermore, beeswax has a versatile role due to its effect on cosmetics’ physical properties, such as hardness, or sensorial qualities, namely color and luster [15].

*Arbutus unedo* L. belongs to the Ericaceae family and is commonly known as “strawberry tree”. Native populations are available in several regions around the world, such as the Atlantic area of western Europe surrounding the Mediterranean Sea [17], the Canary Islands [18], northeastern Africa, and western Asia [19], with Portugal being an important producer [20]. The whole plant possesses a range of qualities, including its ornamental, ecological, and economic significance, along with its therapeutic and medicinal properties. The berries alone present a wide range of bioactive molecules, including sugars, fatty acids, phenolic compounds, fibers, vitamins, minerals, proteins, and carotenoids [19]. Few products containing strawberry tree fruit extract are available on the cosmetics market [21], claiming to reduce the sebum production and boost a matte appearance [22]. Due to their polyphenolic content, berries also exhibit a considerable antioxidant capacity that could be used to rejuvenate and sustain skin’s well-being and combat the oxidative stress related to aging [1].

Conventional and non-conventional methods are traditionally used to obtain polyphenols from natural sources. The non-conventional method offers quicker extraction times, enhanced efficiency, selectivity, and reduction in solvent usage, while adopting alternative solvents and diminishing the presence of harmful substances [8]. Ultrasound-assisted extraction (UAE) is a green methodology widely used for extracting bioactive compounds [8,23]. This technique is influenced by factors such as extraction time, frequency, particle size, solvent concentration, solid-to-liquid ratio, temperature, and ultrasound power. It offers several advantages, including suitability for thermo-sensitive compounds, improved efficiency, faster extraction times, and reduced energy and chemical consumption [8,9,23]. Moreover, the assessment of natural ingredients’ chemical safety is imperative, not only to preserve the consumers’ well-being, but also to uphold the product quality, fulfill the regulatory standards, address ethical and sustainability considerations, advance scientific understanding, and foster consumer confidence in the safety of cosmetic ingredients [24,25]. 

Therefore, this work aims to develop a cosmetic product combining the potent bioactive properties of *A. unedo* berry extract with the protective and healing properties of beeswax. This cosmetic product aims to offer a natural and effective solution for healthcare, while simultaneously enhancing the well-being and economic prosperity of the local communities located in the Natural Park of Montesinho.

## 2. Materials and Methods

### 2.1. Chemicals and Reagents

Chemicals and reagents were bought from Sigma-Aldrich (Steinheim, Germany), Carlo Erba Reagents S.A.S. (Val de Reuil Codex, France), Riedel-de-Haën (Seelze, Germany), Fluka (Seelze, Germany), and Merck (Darmstadt, Germany). The chemicals used for the cosmetic formulation were purchased from Acofarma (Terassa, Spain), Guinama (La Pobla de Vallbona, Spain), butylated hydroxytoluene (BHT) from Fluka (Madrid, Spain), and triethanolamine from Fagron (Terassa, Spain). Cell reagents were provided by PAN-Biotech (Aidenbach, Germany), Invitrogen Corporation (Life Technologies, S.A., Madrid, Spain), VWR International S.A. (Fontenary-sous-Bois, France), and Fisher Chemicals (Geel, Belgium). Human immortalized non-tumorigenic keratinocyte cell line (HaCaT) was purchased from CLS Cell Lines Service (Eppelheim, Germany). The Quick, Easy, Cheap, Effective, Rugged, and Safe (QuEChERS) extraction tubes and cleanup materials were obtained from Agilent Technologies Inc. (Santa Clara, CA, USA), and Z-Sep+ Bulk from Sigma-Aldrich (St. Louis, MO, USA). The Solid Phase Extraction (SPE) column was purchased from Phenomenex (Torrance, CA, USA).

The contaminant screening analyzed the following contaminants: (i) 9 Organochlorine pesticides (OCPs): α- hexachlorocyclohexane (HCH), β-HCH, ζ-HCH, aldrin, endosulfan I, dichlorodiphenyldichloroethane (p,p’-DDD), dichlorodiphenyldichloroethylene (p,p’-DDE), dichlorodiphenyltrichloroethane (o,p’-DDT), and dieldrin; (ii) 5 Pyrethroid pesticides (PYRs): bifenthrin, cyhalothrin 1, cyhalothrin 2, cypermethrin, fenvalerate 1, fenvalerate 2, deltamethrin 1, and deltamethrin 2; (iii) 7 Brominated diphenyl ethers (BDEs): BDE 28, BDE 47, BDE 99, BDE 100, BDE 153, BDE 154, and BDE 183; (iv) 4 Polychlorinated biphenyls (PCBs): PCB 28, PCB 118, PCB 153, and PCB 180; (iv) 6 Orgaphosphorus pesticides (OPPs): dimethoate, chlorpyrifos-methyl, parathion-methyl, malathion, chlorpyrifos, and chlorfenviphos; (v) 7 Organophosphate esters (OPEs): tripropyl phosphate (TPrP), triisobutyl phosphate (TiBP), tri(n-butyl)phosphate (TnBP), tris (2-chloroethyl) phosphate (TCEP), tributoxyethyl phosphate (TBEP), triphenyl phosphate (TPhP), tris (2-ethylhexyl) phosphate (TEHP), and tricresyl phosphate (TCP).

### 2.2. Beeswax

Beeswax was collected from *Apis mellifera* L. bees’ hive apiaries located at the Natural Park of Montesinho (41°53′49″ N, 6°51′58″ W) in October 2022. Samples were packed in sealed plastic bags and stored at −18 °C until further use.

#### 2.2.1. Beeswax Lipid Profile

The fatty acid extraction followed the Folch procedure [26], changing chloroform for dichloromethane, as reported by Ozório et al. [27]. The procedure was performed in triplicate. The samples were diluted (1:20) in dichloromethane:methanol (2:1) 0.01% BHT, vortex-mixed (Analog Vortex Mixer, VWR, Radnor, PA, USA) for 15 min, followed by 10 min of centrifugation (Megafuge 16R centrifuge, Thermo Fisher Scientific, Waltham, MA, USA) at 2500 rpm. The supernatants were filtered through a Whatman n° 1 paper. This process was repeated twice. To clean the filtrate, 0.73% NaCl was added and centrifuged at 2500 rpm for 15 min. The superior part of the supernatant was rejected, and the lower part was filtered through a Whatman n° 1 paper. Lastly, the filtrated was evaporated (R-200, rotavapor system, Buchi, Flawil, Switzerland) in a 40 °C water bath, at approximately 600 mbar. The residue was redissolved in 3 mL of dichloromethane and stored at −20 °C. 

Analysis of fatty acid methyl esters, also processed in triplicate, followed the Bondia-Pons et al. [28] procedure. This procedure is based on a base-catalyzed transmethylation of total lipids. First, 500 µL of the extracted sample was added to 5 mL of sodium methylate (0.5 M), vortex-mixed, and left in a water bath at 100 °C for 10 min, followed by 5 min on ice. Then, 5 mL of boron trifluoride-methanol was added to the mix, heated up to 100 °C for 30 min, followed by 5 min on ice. Next, 1 mL of *n*-hexane with BHT at 0.02% was added and vortex-mixed for 1 min, followed by NaCl and a final centrifuge of 5 min at 3000 rpm. The recovered organic phase was dried with anhydrous sodium sulfate and evaporated (Reacti-Therm III, Thermo Scientific, Waltham, MA, USA) to dryness under nitrogen flow and finally redissolved in *n*-hexane.

For the analysis, a Shimadzu GC-2010 (Kyoto, Japan) with a FID (Flame Ionization Detector) apparatus, equipped with a CP-Sil 88 capillary column of 100 m (0.20 µm film thickness, 0.25 mm inner diameter, Agilent^®^ J&W, Santa Clara, CA, USA) was used. Injections of 1 μL were made in splitless mode, and its port temperature was 260 °C. The temperature was programmed starting at 100 °C and held for 5 min, followed by increases of 8 °C/min to 180 °C (held for 9 min), and then 1 °C/min to 230 °C (held for 1 min). The temperature of the FID was 260 °C. As a carrier gas, Helium (PRAXAIR) at a constant flow rate of 1.18 mL/min was used. The system was operated by GCsolution software (version 2.42.00, Shimadzu Corporation, Kyoto, Japan). Standard solutions were employed for comparison, and identification, of peaks’ retention time. 

#### 2.2.2. Beeswax Carotenoid Profile

To determine the total carotenoid content, the procedure described below was followed. To prepare the extract, the sample (1 g) was extracted with 30 mL of acetone, using a magnetic stirrer (RO 10, IKA, Staufen, Germany). After one hour of extraction, the sample was filtered through a Whatman nº1 paper and later dried under nitrogen flow.

The HPLC system (Shimadzu, Kyoto, Japan) was equipped with a photodiode array detector (PDA) model SPD-M20A. The eluents underwent filtration through a 0.22 µm nylon membrane filter (Fioroni Filters, Ingré, France) with a vacuum pump (Dinko D-95, Barcelona, Spain) and were degassed for 15 min in an ultrasonic bath (Sonorex Digital 10P, Bandelin DK 255P, Berlin, Germany). Samples were injected on an analytical HPLC with a low-pressure quaternary pump (model LC-20AT), a degasser (model DGU-20A5R), an auto-sampler (model SIL-20AT), and a column oven (model CTU-20AC). Separation followed the process described by Fernandes et al. [29] with some adjustments. For the carotenoid’s separation, a C30 YMC Carotenoid S-5 µm (25.0 × 0.46 cm; 5 µm particle size) column (Kyoto, Japan) was used. The solvent system consisted of a mixture of methanol and methyl tert-butyl ether. The solvent system started with 95% methanol, followed by a gradient to obtain 70% methanol at 30 min, then 50% methanol at 60 min, and, finally, at 65 min, 95% methanol. The solvent flow rate was 0.9 mL/min. Chromatograms were recorded at 450 nm, and LCsolution software (version 2.1. Shimadzu Corporation, Kyoto, Japan) processed the data. The carotenoids were identified through a comparison of their UV–Vis spectra and retention times with the standards injected in the same conditions.

β-Carotene curve: y = 1.33 × 10^8^x − 8.61 (*R*^2^ = 0.999; LOD = 1.18 × 10^−7^ mg/mL; LOQ = 3.57 × 10^−7^ mg/mL)

Lutein curve: y = 1.33 × 10^8^x − 8.75 × 10^4^ (*R*^2^ = 0.997; LOD = 1.29 × 10^−6^ mg/mL; LOQ = 3.90 × 10^−6^ mg/mL)

Zeaxanthin curve: y = 2.97 × 10^8^x + 2.47 × 10^4^ (*R*^2^ = 0.991; LOD = 3.47 × 10^−6^ mg/mL; LOQ = 1.05 × 10^−5^ mg/mL)

#### 2.2.3. Beeswax Contaminant Screening Analysis

For the contaminant screening of the sample, the previously described procedure was followed, namely a QuEChERS method with d-SPE clean-up [30]. The QuEChERS extraction tubes used contained the following composition: 1.0 g sodium chloride, 4.0 g magnesium sulfate, 1.0 g sodium citrate, and 0.5 g sodium hydrogencitrate sesquihydrate. The clean-up used contained the following composition: 50 mg PSA, 50 mg C18EC, and 150 mg magnesium sulfate. In addition to these, it also used Z-Sep+ Bulk. 

In the first instant, a QuEChERS extraction procedure was followed: into a 50 mL falcon tube, 5 mg of beeswax was weighed, 10 mL of acetonitrile was added, and it was vortex-mixed for 1 min. Then, the QuEChERS extraction kit was added to the resulting mix, and vortex-mixed for 1 min. After the mix was centrifugated at 4500 rpm for 5 min, the supernatant was put into a 2 mL tube containing the cleanup. Afterwards, the tube was vortex-mixed for a minute and centrifuged for 5 min at 4500 rpm. 

The use of the cleanup was intended to further clean the sample of matrix interferents, like pigments and other non-volatile substances [31]. Since the supernatants were not clear, the cleanup did not remove all the interferents. Therefore, we needed to proceed again with this step.

In a second run, the procedure described above was repeated, with the addition of 25 mg of Z-Sep+ Bulk to the cleanup. After the final centrifugation, the supernatant of the beeswax sample was extracted and dried into nitrogen. Finally, it was redissolved in the same volume of *n*-hexane. The vials with the beeswax sample were later inserted into the respective gas chromatography (GC).

For the analysis of the OCPs, PYRs, PCBs, and BDEs, a Shimadzu GC-2010 with an ECD (Electron Capture Detector) apparatus was equipped with a Zebron 5MSi capillary column of 30.0 m (0.25 µm film thickness, 0.25 mm inner diameter, Phenomenex, Torrance, CA, USA). Injections of 1 μL were made in splitless mode, and its port temperature was 250 °C. The temperature started at 40 °C and was held for 1 min, followed by increases of 15 °C/min to 120 °C (held for 1 min), then 10 °C/min to 150 °C (held for 1 min), then 5 °C/min to 180 °C (held for 1 min), then 5 °C/min to 200 °C (held for 1 min), and finally 5 °C/min to 290 °C (held for 21 min). The ECD temperature was 300 °C. As a carrier gas, helium was used at a constant flow rate of 1.00 mL/min. The system was operated by GCsolution software (version 2.42.00, Shimadzu Corporation, Kyoto, Japan).

For the analysis of the OPEs and OPPs, a Shimadzu GC-2010 with an FPD (Flame Phototometric Detector) apparatus was equipped with a Zebron 5MSi capillary column of 30.0 m (0.25 µm film thickness, 0.25 mm inner diameter). Injections of 1 μL were made in splitless mode, and its port temperature was 250 °C. The temperature started at 80 °C and was held for 1 min, followed by increases of 10 °C/min to 170 °C (held for 1 min), then 10 °C/min to 200 °C (held for 0.5 min), then 1 °C/min to 202 °C (held for 1 min), then 0.05 °C/min to 203 °C (held for 1 min), then 4 °C/min to 210 °C (held for 0 min), then 10 °C/min to 270 °C (held for 0 min), then 2 °C/min to 276.5 °C (held for 1 min), then 1 °C/min to 278 °C (held for 1 min), and finally 1 °C/min to 280 °C (held for 1 min). The temperature of the FPD was 300 °C. As a carrier gas, helium was used at a constant flow rate of 0.93 mL/min. The system was operated by GCsolution software (version 2.42.00, Shimadzu Corporation, Kyoto, Japan).

### 2.3. Arbutus unedo *L.*

Strawberry tree fruits were collected at the Natural Park of Montesinho (41°53′49″ N, 6°51′58″ W) in October 2022. The fruits were lyophilized (Cryodos-80, Telstar, Barcelona, Spain), milled with a mortar and pestle, and stored at room temperature until further extraction.

#### 2.3.1. Ultrasound-Assisted Extraction

The extraction followed the procedure described by Silva et al. [32], with minor modifications. The lyophilized powdered fruit was extracted with water (1:20) at different times (15, 30, 45, 60, or 90 min), employing an ultrasonic intensity (Sonics Vibra Cell, model VCX50, Sonics and Materials Inc., Newtown, CT, USA) of 30 W/m^2^. Afterward, the extracts were centrifuged at 5000 rpm for 15 min at 20 °C, and the supernatants were filtered through a Whatman n° 1 paper and subsequently frozen at −80 °C until lyophilization.

#### 2.3.2. Determination of the Total Phenolic Content (TPC)

The TPC was determined following the Folin-Ciocalteu procedure described by Singleton and Rossi [33], with minor modifications [34]. The assays were performed in triplicate, and gallic acid was used as standard (linearity range = 5–100 μg/mL; y = 0.008x − 0.040; *R*^2^ = 0.998; LOD = 6.62 mg GAE/g dw; LOQ = 22.09 mg GAE/g dw). Results were expressed as mg of gallic acid equivalents (GAE) per gram of extract on dry weight (dw) (mg GAE/g dw).

#### 2.3.3. In Vitro Antioxidant/Antiradical Activities

##### Ferric Reducing Antioxidant Power (FRAP) Assay

The FRAP assay was performed based on the Benzie and Strain procedure [35], with minor modifications. Ferrous sulphate was used as standard (linearity range = 25–500 μg/mL; y = 0.002x + 0.017; *R*^2^ = 0.997; LOD = 49.53 µmol FSE/g dw; LOQ = 165.10 µmol FSE/g dw). Results were expressed as µmol of ferrous sulphate equivalents (FSE) per gram of extract on dw (µmol FSE/g dw).

##### ABTS Radical Scavenging Assay

The ABTS radical scavenging assay was performed based on the Re et al. procedure [36], with minor modifications. Ascorbic acid was used as standard (linearity range = 5–50 μg/mL; y = 0.011x + 0.060; *R*^2^ = 0.999; LOD = 1.57 mg AAE/g dw; LOQ = 5.24 mg AAE/g dw). Results were expressed as mg of ascorbic acid equivalents (AAE) per gram of extract on dw (mg AAE/g dw).

##### DPPH Free Radical Scavenging Assay

The DPPH free radical scavenging assay was performed according to Barros et al. [37], with minor modifications. Trolox was used as standard (linearity range = 5–75 μg/mL; y = 0.007x + 0.062; *R*^2^ = 0.996; LOD = 8.30 mg TE/g dw; LOQ = 27.70 mg TE/g dw). Results were expressed as mg of Trolox equivalents (TE) per gram of extract on dw (mg TE/g dw).

#### 2.3.4. Reactive Oxygen Species (ROS) Scavenging Capacity

Hypochlorous acid (HOCl), superoxide (O_2_^•−^), and peroxyl (ROO^•^) radical scavenging assays [38] were tested. The assays were performed in triplicate and following the methodologies described below.

##### Hypochlorous Acid Scavenging Assay

The HOCl quenching capacity was determined based on the protocol described by Gomes et al. [38]. Catechin (3.91 to 0.122 μg/mL) and gallic acid (0.488 to 15.625 μg/mL) were used as positive controls. The results were presented as the inhibition of the HOCl-induced oxidation of dihydrorhodamine 123 (DHR), expressed as IC_50_ (minimum inhibitory concentration to reduce the activity by 50%).

##### Superoxide Radical Scavenging Assay

The superoxide (O_2_^•−^) capacity was determined based on the protocol described by Gomes et al. [38]. Catechin (7.81 to 250 μg/mL) and gallic acid (1.95 to 62.5 μg/mL) were used as positive controls. The results were expressed as IC_50_.

##### Peroxyl Radical Scavenging Assay

The peroxyl radical (ROO^•^) scavenging assay was performed following the protocol described by Gomes et al. [38]. Catechin (0.00625 to 0.2 μg/mL) and gallic acid (0.046875 to 1.5 μg/mL) were used as positive controls, and Trolox (0.046875 to 1.5 μg/mL) was used as a standard. The results were expressed as µmol of Trolox equivalents per microgram of extract on dw (µmol TE/mg dw).

#### 2.3.5. Polyphenolic Profile

The study of the polyphenolic profile of *A. unedo* L. extracts was acquired through a HPLC-PDA system, following the procedure described by Moreira et al. [39]. The extracts were diluted in water and methanol (80:20), and filtered using a 0.22 µm nylon filter. The column used was a Gemini C18 column (250 mm × 4.6 mm, 5 µm, Phenomenex, Torrance, CA, USA) kept at 25 °C, and the mobile phase was a mixture of water and methanol with formic acid (0.1%). The eluent, before being introduced into the HPLC-PDA system, went through a degassing process. For the identification of the compounds, LCsolution software (version 2.1, Shimadzu Corporation, Kyoto, Japan) was used, where the retention time and the UV-Vis spectra of the individual phenolic compounds were compared with the reference standard chromatograms obtained under the same conditions. The content of each phenolic detected was expressed as mg of compound per 100 g of extract on dw (mg compound/100 g dw).

#### 2.3.6. Anthocyanins Determination

The anthocyanin content was determined by HPLC-PDA, as described by Nile et al. [40]. The mobile phase was composed of methanol with 0.5% formic acid (A) and water with 5% formic acid (B) at a flow rate of 1.0 mL/min. The gradient used was: 0–11 min: 7–10.5% A; 11–18 min: 10.5–13% A; 18–21 min: 13–16% A; 21–38 min: 16–21% A; 38–61 min: 21–35% A; and 61–63 min: 35–100% A, followed by 100% A for 4 min and back to 7% A in 3 min and 5 min of reconditioning before the next injection. The injection volume was 20 µL and the column temperature was 40 °C. The calibration curves were obtained by injecting mixtures of the individual standards in the concentration range of 1–50 mg/L, and the quantification of individual anthocyanins was made at 520 and 280 nm, depending on the compound maximum absorption.

#### 2.3.7. Carotenoid Profile

The determination of the carotenoid profile of the strawberry tree extract followed the same procedure as described in Section 2.2.2. Here, 1 g of the optimized extract was obtained with 20 mL of acetone.

#### 2.3.8. Contaminants Screening Analysis 

The contaminants screening of *A. unedo* berry extracts utilized a 500 mg C18-E column, following the procedure described in [41], with minor modification. Initially, 10 mg of extract was diluted in 1 mL of ultrapure water and vortex-mixed for 1 min. Then, the column was conditioned in the manifold, and the solvents (of dichloromethane:ethyl acetate (1:1), methanol, and ultrapure water) were conditioned (2 × 2 × 2) twice through the column. Next, 1 mL of the sample was filtered through the column and followed by 6 mL of ultrapure water. After drying the cartridge for 20 min, the sample was eluted with 1 mL of dichloromethane: ethyl acetate (1:1). The extracted solution was dried under nitrogen flow and then redissolved in *n*-hexane. A vial containing the solution was later inserted in the respective GC. 

For the analysis of the POPs, pyrethroids, and OPEs, the procedure described in Section 2.2.3 was followed. 

#### 2.3.9. Cell Viability Assays

Cell viability assays were performed on keratinocytes (HaCaT) to evaluate the cytotoxic potential of strawberry tree extracts. Passages 80–88 of cells were used. The cell line was cultivated in Dulbecco’s Modified Eagle Medium (DMEM) supplemented with 5% fetal bovine serum, 1% non-essential amino acids, and 1% Penicillin-Streptomycin. Cells were incubated in a 5% CO_2_ environment at 37 °C (ESCO GB Ltd., Barnsley, UK). The vital mitochondrial dye 3-(4,5-dimethylthiazol-2-yl)-2,5-diphenyltetrazolium bromide (MTT) assay was performed following the methodology described by Pinto et al. [42]. Triton X-100 1% (*w*/*v*) and DMEM were used as negative and positive controls, respectively. Results were expressed as percentages (%) of cell viability.

### 2.4. Cosmetic Product Development

#### 2.4.1. Formulation of a Hydrophilic Cream

Table 1 summarizes the composition of the oil-in-water (o/w) cream formulation. Briefly, the oil phase ingredients were heated using a water bath (Nahita, Auxilab, Navarra, Spain) at 80–90 °C. The ingredients of the aqueous phase were mixed and heated at the same temperature as the oil phase. Once both phases reached the same temperature, the aqueous phase was slowly added to the oil phase with continuous stirring. The mixture was then stirred until cooled to room temperature. To improve the formulation’s consistency, Carbopol^®^ 940 was added, and triethanolamine was used to adjust the pH. After optimizing the formulation, 10% of *A. unedo* extract was incorporated. Both formulations, with (Y) and without (X) extract, were stored at 25 °C and evaluated for accelerated stability, pH, color, texture, and rheology.

#### 2.4.2. Formulation Characterization

##### Accelerated Stability by Centrifugation

Approximately 7 mL of both formulas was placed in each centrifuge tube and submitted to a cycle of centrifugation (Centrifuge 5804, Eppendorf, Berzdorf, Germany) for 30 min at 3000 rpm. This evaluation was made immediately after preparing the cream.

##### pH

To determine the formulation pH, a pHmeter (Basic20, Crison Instruments, Barcelona, Spain) was used with a built-in probe, previously calibrated. 

##### Color

The formulation color was evaluated using a colorimeter (Chroma meter CR-400, Konica Minolta, Tokyo, Japan). The data were processed by the Spectra Magic NX software (version 2.81, Konica Minolta, Tokyo, Japan). The colorimeter was previously calibrated on a white surface (standard). The parameters evaluated were the L* and the chroma (C*) parameter, which represents the color of the product. L* represents the light component, ranging from 0 (black) to 100 (white), whereas a* gives the chromatic scale between green (−a*) and red (+a*), and b* values represent the chromatic scale between blue (−b*) and yellow (+b*) (41). Equation (1) represents the formula to calculate the C* parameter.

(1) $$C^{*}=\sqrt{\left({a^{*}}^{2}+{b^{*}}^{2}\right)}$$

##### Texture

The texture profile was determined using a texture analyzer (TA-XT2i, Stable Micro Systems, Surrey, UK) and the data were processed by Texture Exponent software (version 6.1.26.0, Stable Micro Systems, Surrey, UK). Adhesiveness and firmness were evaluated. A 25-mm Perspex cylindrical probe was used. The test was performed in compression mode, with a 5 kg loading cell, a trigger force of 0.049 N, a penetration depth of 5 mm, and a test speed of 3 mm/s. The force versus distance data were graphed, enabling the determination of adhesiveness (as the negative area) and firmness (as the maximum force) of the formulation.

##### Rheology

The rheological characteristics of the formulations were evaluated using a rotational rheometer (Kinexus-lab+, Malvern Instruments; Worcestershire, UK) with the PU20 SR4367 SS probe, and the geometry mode applied was plate-to-plate with a 1 mm gap. The software used was rSpace software for Kinexus (version 2.0.0.0, NETZSCH-Gerätebau GmbH, Selb, Germany).

Viscosity

The formulation viscosity was evaluated using the following conditions: a shear rate between 0.1 and 100 s^−1^, and 10 samples per decade.

Thixotropy

To evaluate the formulations’ thixotropy, a tree phase test was applied, with the following conditions. The operation started with a shear rate of 0.1 s^−1^ for 60 s, then the shear rate was increased up to 100 s^−1^ for 30 s, and finally, the shear rate was reduced for 120 s to 0.1 s^−1^.

Amplitude Sweep Test

To determine the linear viscoelastic region, after preparation the following conditions were considered: a shear strain between 0.1 and 100 s^−1^, a frequency of 1 Hz, and 10 samples per decade.

##### Long-Term Stability Evaluation

Both formulations were evaluated in triplicates, at time 0 (T0), after 60 days (T60), and at room temperature. The parameters analyzed were pH, color, texture, viscosity, and thixotropy.

### 2.5. Statistical Analysis

The results were reported as mean ± standard deviation (SD). IBM SPSS Statistics 27.0 software (SPSS Ins, Chicago, IL, USA) was employed to perform the data’s statistical analysis. To investigate the differences between samples for all assays and compare the means, the one-way ANOVA test and Turkey’s HSD test were applied, respectively. For *p*-value < 0.05, samples were considered statistically significant. 

Microsoft Office Excel (2016) was applied to calculate the IC_50_ values, as well as the standard error or mean values of antiradical assays. The software was also used to calculate the means and standard deviation in the formulation characterization. 

GraphPad Prism 9.0.2 software (GraphPad Software, La Jolla, CA, USA) was used to determine the area under the curve on the ORAC assay.

## 3. Results and Discussion 

### 3.1. Beeswax

#### 3.1.1. Lipid Profile

Table 2 shows the detailed fatty acid profile (%) of beeswax. A total of 14 fatty acids were identified, with saturated fatty acids (SFA) presenting the predominant percentage (74.10 ± 1.01 × 10^−2^%). Regarding unsaturated fatty acids (UFAs), beeswax holds a higher value of omega-6 (ω6). The main fatty acids present in beeswax were palmitic (C16:0), oleic (C18:1 *n*-9c), and linoleic (C18:2 *n*-6).

The method’s precision was evaluated on the same day (repeatability) over three replicates, with RSD values being below 20%. 

Studies on the fatty acid composition of beeswax have shown a predominance of SFA with even numbers of carbon atoms between C20 and C36 [11]. However, some authors have reported the presence of SFA with odd numbers of carbon atoms, like C21:0 and C23:0 (127). The predominance of SFA, namely fatty acids C16:0 and C24:0, has also been confirmed by various authors [14,43,44]. A study by Jiménez et al. [43] on Spanish beeswax showed that the beeswax contained 16 SFA and 13 UFAs. Unlike our sample, it did not contain C10:0, but included additional fatty acids, such as C15:0, C21:0, C23:0, even-chain acids between C26:0 and C36:0, C18:1t, and even chain acids between C20:1 and C36:1 [43]. A variation in fatty acid composition between hives has already been described [45]. Given these differences, a comprehensive fatty acid analysis for each hive wax is recommended for cosmetic applications.

In cosmetics, fatty acids can be used as brighteners, emulsifiers, and softening agents, influencing the skin’s normal function [46]. Fatty acids are a key component of the *stratum corneum*, where nearly 50% of the free fatty acids are saturated [47]. In *stratum corneum* membranes, palmitic, stearic, and lignoceric acids are present, along with lower concentrations of oleic, linoleic, and linolenic acids [48]. SFA helps reduce transepidermal water loss (TEWL) and minimize irritation, in contrast to the effects of UFAs [47].

#### 3.1.2. Contaminant Screening Analysis 

**Figure 1:**
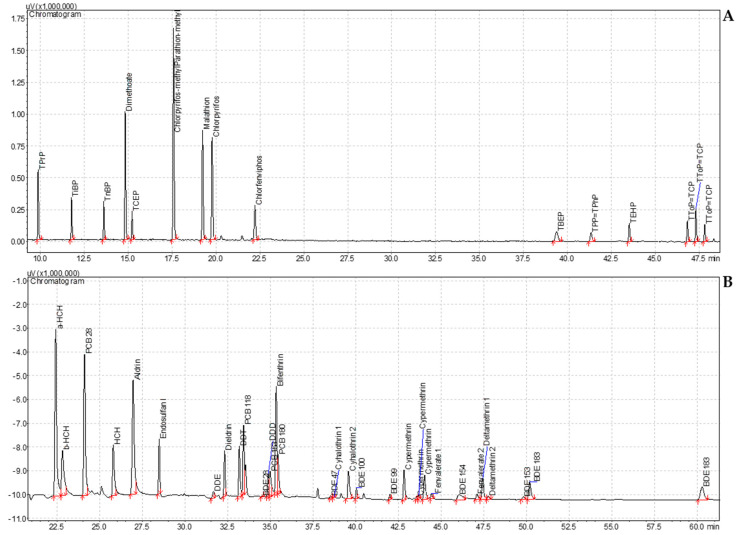
(**A**) Chromatogram of 50 μg/L of OPEs (organophosphate esters) and OPPs (orgaphophorus pesti-cides) in *n*-hexane; (**B**) chromatogram of 25 μg/L of OCPs (organochlorine pesticides), PYRs (pyre-throids), PCBs (polychlorinated biphenyls), and BDEs (brominated diphenyl ethers) in *n*-hexane.

The contaminants screening analysis results are summarized in Figure 1. The obtained chromatograms were used for later analyses of beeswax and *A. unedo* berry extracts.

**Figure 2:**
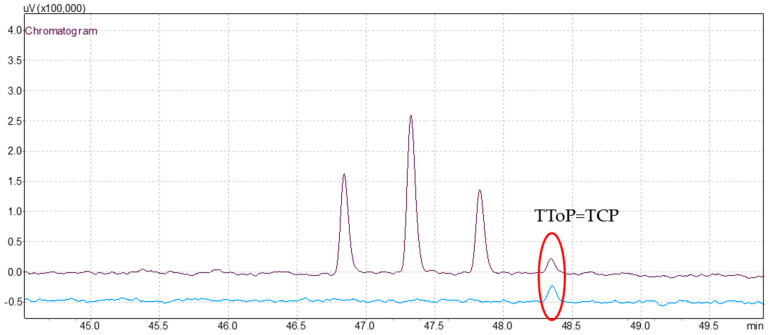
Comparison of 50 μg/L of OPEs and OPPs chromatogram (black), and beeswax sample (blue). Identification of tricresyl phosphate (TCP) peak (red).

After extraction and injection of beeswax extract, a screening analysis was conducted using both, and the results were compared with those of standard solution chromatograms (Figure 1). Qualitative analysis through chromatogram comparison indicated the presence of one detectable peak. Figure 2 shows the comparison between chromatograms and the identification of the respective peak. 

By comparing the chromatograms based on retention times, the detected contaminant was identified. Quantification of the contaminant was carried out after calibration. Table 3 presents the identification and quantification of the peak identified in the beeswax sample, along with the corresponding analytical parameters.

In the beeswax extract, the peak detected at 48.165 min corresponds to TCP (an OPE). The concentration of TCP in the beeswax extract was 8.38 µg/L, however, this is not reported in Regulation (EC) No 1223/2009 of the European legislation [49]. This highlights an important point, the need for continuous update to the regulations. Nowadays, new emerging pollutants and their associated risks are identified, therefore, it is crucial to incorporate them in safety assessments. This proactive strategy addresses emerging risks to be proactively addressed and preventive measures to be implemented. 

### 3.2. Arbutus unedo *L.* Extract

#### 3.2.1. Chemical and Bioactivity Characterization

Table 4 summarizes the TPC and in vitro antioxidant/antiradical activities of *A. unedo* berry extracts.

As can be observed in Table 4, across all assays, the 60-min extract achieved the highest results, while the 15-min extract obtained the lowest ones.

Regarding the ABTS^•+^ scavenging assay, results ranged from 26.78 to 30.36 mg AAE/g dw. A clear correlation between extraction time and antioxidant activity was evident, with the 15-min extract showing significant differences (*p* < 0.05) when compared to the 30-, 60- and 90-min extracts. These results were significantly higher than the ones reported by Zitouni et al. [50], who reported an antioxidant capacity between 2.25 and 19.57 mg AAE/g dw in *A. unedo* Moroccan berries. The differences observed may be due to the berries’ origin, and the conventional method employed (solvent extraction with ethanol). 

In the DPPH^•^ scavenging assay, the values varied between 37.62 and 43.83 mg TE/g dw. Here, the 60-min extract showed significant differences (*p* < 0.05) when compared to the 15-, 45-, and 90-min extracts. Izcara et al. [51] studied the radical scavenging activity of unripe *A. unedo* Spanish berries, harvested over different years in the Madrid and Extremadura regions (Spain), which reported higher values (2018—42.81 mg TE/g dw; 2016—74.76 mg TE/g dw; 2015—295.31 mg TE/g dw). Nevertheless, these results were obtained using a different method—solvent extraction with methanol—and from berries harvested in different locations. 

As far as the FRAP assay is concerned, results varied between 353.69 and 415.61 µmol FSE/g dw. The 60-min extract presented significant differences (*p* < 0.05) when compared to the other four extracts, while the 30-min extract showed significant differences (*p* < 0.05) with the 15-, 45-, and 90-min extracts.

The TPC results ranged from 24.69 to 30.27 mg GAE/g dw. The 60-min extract showed significant differences (*p* < 0.05) when compared to all other extracts, apart from the 30-min extract. On the other hand, the 30-min extract showed significant differences (*p* < 0.05) with the 15- and 45-min extracts. Zitouni et al. [50] reported a TPC of 30.98 mg GAE/g dw for *A. unedo* Moroccan berries, consistent with the findings of this study. Mendes et al. [52] and Barros et al. [53] studied the TPC of *A. unedo* Portuguese berries collected at the Natural Park of Montesinho, reporting values of 16.7 and 126.83 mg GAE/g dw, respectively. Despite being from the same origin, the differences may be explained by the different extraction methods used, such as boiling water [52] and methanolic extraction [53]. Izcara et al. [51] also studied the TPC of unripe *A. unedo* Spanish berries from different crop years, reporting higher values (2018—34.73 mg GAE/g dw; 2016—26.71 mg GAE/g dw; 2015—36.12 mg GAE/g dw). Overall, the ultrasonic extraction method proved more efficient and yielded better results.

As far as the ROS scavenging capacities are concerned, namely against HOCl, O_2_^•−^, and ROO^•^, Table 5 summarizes the results obtained.

The HOCl and the O_2_^•−^ scavenging capacities were expressed as IC_50_, meaning the in vitro concentration needed to decrease the reactivity of the species tested by 50%. Catechin and gallic acid were used as positive controls. The HOCl scavenging varied between IC_50_ = 17.23 µg/mL (15-min extract) and IC_50_ = 31.63 µg/mL (30-min extract). Although the 60-min extract did not show the lowest value, there were no significant differences (*p* > 0.05) when compared with the 15-min extract. To the best of our knowledge, no studies regarding the HOCl scavenging assays for *A. unedo* berry extract are available in the literature. For the O_2_^•−^ scavenging assay, the values ranged from IC_50_ = 90.51 µg/mL (60-min extract) to IC_50_ = 342.44 µg/mL (15-min extract). The 60-min extract showed significant differences (*p* < 0.05) when compared to all other extracts, as well as the 15- and 30-min extracts. A study conducted on *A. unedo* Algerian berries extracts reported an IC_50_ = 84 µg/mL [54], considerably higher than the one attested for the Portuguese fruits. As can be observed, for both assays, the results presented significant differences (*p* < 0.05) with the positive controls used. 

For the ROO^•^ scavenging assay, the results ranged from 0.11 (30-min extract) to 0.23 (15-min extract) µmol TE/mg dw. No significant differences (*p* > 0.05) were noted between the different extracts. Boulanouar et al. [54] also studied the ROO^•^ scavenging capacity of *A. unedo* Algerian berries extracted by sonication, reporting a lower value (0.09625 µmol TE/mg dw). Moreover, a study on *A. unedo* Portuguese berries collected from Arrábida Natural Park, a southern region of Portugal, reported a similar ROO^•^ scavenging activity (0.1166 µmol TE/mg dw) [55]. Similar to the other ROS, the results were significantly different from the positive controls used (*p* < 0.05).

The phytochemical profile of each extract was also studied through HPLC-PDA analysis. Table 6 summarizes the identification and quantification of the phenolic compounds detected.

As reported in Table 6, the total phytochemical content varied depending on the different extraction times, with the 45-min extract holding the higher amount (2574 mg/100 g dw), followed by the 60-min (2502 mg/100 g dw), 90-min (2336 mg/100 g dw), 30-min (2191 mg/100 g dw), and 15-min (1891 mg/100 g dw) extracts. Overall, the main group identified was phenolic acids, followed by flavan-3-ols, tannins, flavonols, anthocyanins, and stilbenes. 

Within all extracts, the most abundant compound was gallic acid, with the 45-min extract showing the higher concentration (2238 ± 112 mg/100 g dw), followed by the 60-min, 90-min, 30-min, and 15-min extracts. Following, a significant presence of catechin and its derivatives (epicatechin, (-)-epigallocatechin, and (-)-gallocatechin), as well as procyanidin B2, was found in all samples. In addition, the presence of quercetin-3-*O*-galactoside and 4-*O*-caffeoylquinic acid across all extracts should be highlighted.

Despite some differences found in quantitative and qualitative profiles, which could be explained by the sample’s origin, the results align with reports found in the literature. Izcara et al. [51] identified 18 phenolic compounds in *A. unedo* Spanish berries, with catechin, procyanidin B2, and epigallocatechin being the most abundant. Zitouni et al. [50] analyzed the polyphenolic profile of *A. unedo* Moroccan berries, identifying 17 compounds across five genotypes, with, according to the authors, the main compounds being gallocatechin, catechin, gallic acid, ellagic acid, and chlorogenic acid. Ayaza et al. [56] reported the presence of gallic, gentisic, protocatechuic, *p*-hydroxybenzoic, vanillic, and *m*-anisic acids in *A. unedo* berries. Pimpão et al. [57] also confirmed the presence of phenolic compounds in *A. unedo* berries collected from the Alentejo region, with lower gallic acid concentrations (117.2 mg/100 g dw) and (+)-catechin (25.46 mg/100 g dw). Guimarães et al. [58] identified cyanidin 3-*O*-glucoside (11.40 µg/100 g dw), cyanidin 3-*O*-pentoside (1.45 µg/100 g dw), and delphinidin 3-*O*-glucoside (0.91 µg/100 g dw) in ethanolic extracts of *A. unedo* berries collected from the Natural Park of Montesinho, with concentrations significantly lower than the ones observed in the present study (for delphinidin 3-*O*-glucoside and cyanidin 3-*O*-glucoside).

Phenolic compounds are usually associated with strong antioxidant activity [17,58]. In 2014, Hwang et al. [59] reported that the reduction of ROS production promotes type I procollagen expression in cells treated with gallic acid after UVB exposure, suggesting that gallic acid could potentially prevent skin photoaging [59]. Moreover, gallic acid may also inhibit melanin production, making it a potential ingredient against hyperpigmentation skin diseases [60]. Bae et al. [61] attested the capacity of catechin against UV radiation effects, while Yoshino et al. [62] demonstrated the photoprotective effect of emulsions with catechin on a 3D-cultured human skin model after UV exposure.

Carotenoids were not identified in beeswax extract, even after sample concentration. The same occurred for the *A. unedo* berries’ 60 min of aqueous extract. 

#### 3.2.2. Cell Viability Assays

The effects of the different extracts on keratinocytes’ viability were assessed by an MTT assay (Table 7).

The 60-min extract, at all tested concentrations, showed viabilities higher than 90%. For this extract, the values decreased in the following order of concentration: 125 µg/mL (105.36%) < 250 µg/mL (99.16%) < 500 µg/mL (93.34%) < 1000 µg/mL (90.17%). To the best of our knowledge, this is the first study that evaluated the effects of *A. unedo* berry extract on keratinocyte viability. Nevertheless, a study using human neuroblastoma (SK-N-MC) cell line concluded that concentrations of *A. unedo* fruit extracts above 125 µg GAE/mL led to a decrease in cell viability [55]. Bebek Markovinović et al. [63] studied the biological effects of *A. unedo* berry extracts on different human carcinoma cell lines. According to the authors, a reduction in the viability of gastric cancer (AGS) and hepatocarcinoma (HepG2) cell lines was observed after exposure to a concentration of 500 µg GAE/mL.

#### 3.2.3. Contaminant Screening Analysis 

Following the extraction and injection of five *A. unedo* berry extracts into both GCs, a screening analysis was performed. The chromatograms obtained were compared with the standard solution chromatograms (Figure 1). A qualitative analysis of the chromatograms revealed that four extracts were considered clean from the tested contaminants, while the 15-min extract displayed a distinct peak. 

**Figure 3:**
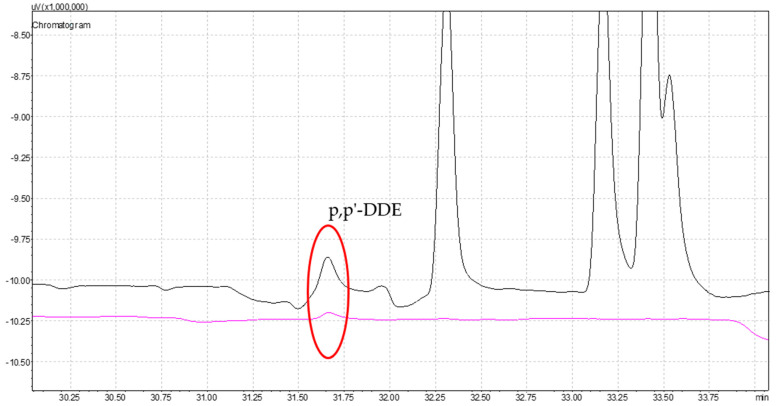
Comparison of 25.0 μg/L of OCPs chromatogram (black) and *A. unedo* berries’ 15-min extract (pink). Identification of p,p’-DDE peak (red).

Figure 3 illustrates the chromatogram comparisons and the identification of the corresponding peak.

Table 8 provides the identification and quantification of the peak detected in the 15-min extract, along with the corresponding analytical parameters.

The peak detected at 31.6 min corresponds to p,p’-DDE, which is below the LOD, indicating the absence of this compound in the extract. Since p,p’-DDE is also not listed in the existent Annex II of Regulation (EC) No 1223/2009 of the European legislation [49], this sample can be considered safe for cosmetic use within the context of the studied groups of compounds. This highlights the importance of regular regulatory updates to ensure the compliance with evolving safety standards.

To the best of our knowledge, no data were available regarding the presence of p,p’-DDE in *A. unedo* samples. However, a study conducted on roots and leaf vegetables reported the presence of DDE in average concentrations of 12.3 and 13 ng/g, respectively [64]. Moreover, it is available in studies that have evaluated the presence of pesticides considered by Regulation (EC) No 1223/2009 in samples for cosmetic use. Silva et al. [41] reported vestigial levels (in the range of 8–9 ng/g) of β-endosulfan and p,p’-DDE in *Salicornia ramosissima*, while Fernandes et al. [65] did not detect OCPs or OPPs in different traditional medicinal plants, which are often contaminated with a wide range of pesticides [66].

### 3.3. Formulation Characterization

#### 3.3.1. Accelerated Stability

The accelerated stability test allows for the analysis of the formulation’s behavior when subjected to variations that may occur during its period of usage, namely coalescence, sediment formation, and phase separation, among others [67]. 

Before pH adjustment with triethanolamine, phase separation occurred due to the low pH. After pH adjustment, the creams maintained their integrity as the pH increased close to neutrality, which is required for Carbopol^®^ 940 gelation [68].

#### 3.3.2. pH

Skin naturally presents an acidic pH, typically ranging from 4.1 to 5.8, with some exceptions [69]. This value is also dependent on external and physiological factors, such as age [69,70]. Given the widespread use of topical formulations, pH evaluation is a key aspect to ensure that formulations do not affect the skin barrier [69]. Table 9 presents the different parameters analyzed during formulas’ X and Y storage.

As shown, the pH values of both formulations were between 6 and 7. Over time, at room temperature, both formulations experienced a slight increase in pH, with no significant differences (*p* > 0.05).

These results are consistent with those of Leite et al. [3] who produced an emulsion containing goji berry extract and did not observe significant differences over 30 days. Similarly, Pinto et al. [5] reported a comparable outcome for an o/w semi-solid formulation incorporating *C. sativa* shell extract, attesting that the presence of an antioxidant extract helped maintain stable pH values over 30 days. Cefali et al. [4] developed o/w emulsions incorporating berry (blackberry and raspberry) extracts, and reported that the pH remained stable over fifteen consecutive days.

#### 3.3.3. Color

The measurement of color in cosmetic products allows the assessment of stability over time during storage. In this study, the colorimetric parameters were evaluated over a 60-day storage period at room temperature (Table 9).

Considering lightness, both formulations exhibited high values, ranging from 82.96 (formula Y) to 84.10 (formula X), concluding that the extract addition led to a slightly darker formulation. Regarding the C* parameter, values varied from 7.81 (formula X) to 11.41 (formula Y), with formula Y presenting a higher b* and lower a* value. Significant differences (*p* < 0.05) were observed in lightness, redness, and yellowness, with both presenting a lightness decrease, while redness and yellowness increased. 

Similarly, a semi-solid formulation prepared by Pinto et al. [5] incorporating *C. sativa* shell extract registered a decrease in L* parameter through time, alongside an increase in redness and yellowness. Almeida et al. [6] produced a hydrogel containing *C. sativa* leaf extract and demonstrated that, after a month of storage, the L* and b* parameters remained unchanged, while a decrease in a* parameter was observed. Moreover, a hydrogel containing *C. sativa* bur extract presented a slight increase in the L* parameter, with no significant differences in redness and yellowness [7].

#### 3.3.4. Texture

Texture reflects the physical characteristics of a product, perceived by touch during application [70]. This parameter can be characterized by firmness and adhesiveness, which are assessed by the deformation of the product when a certain force is applied. Firmness is the force required to cause deformation and is associated with the application of the product on the skin, while adhesiveness is the work done to overcome the attractive forces between the surface of the probe and the surface of the sample, being related to adhesion [71,72]. 

**Figure 4:**
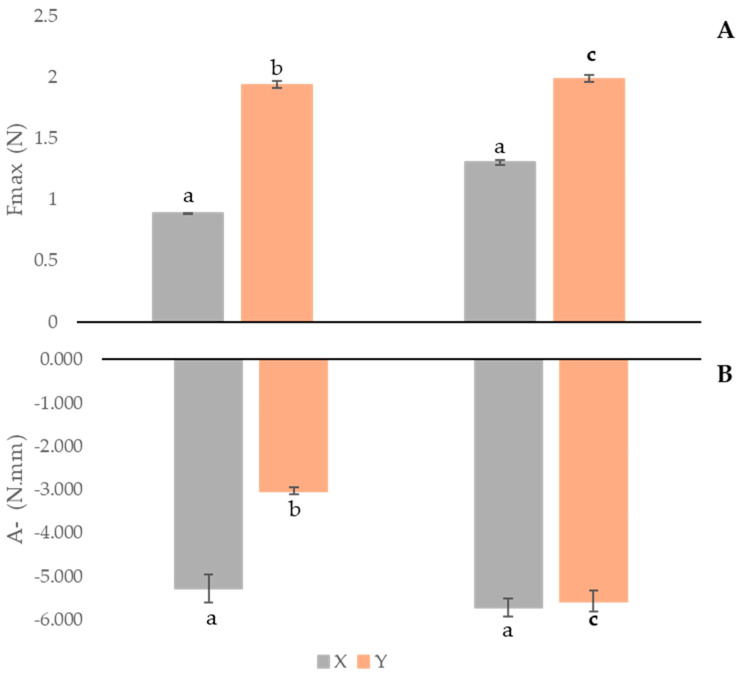
Comparison of firmness (**A**), and adhesiveness (**B**), over 60 days of formulas X: cream without the *A. unedo* extract; and Y: cream containing 10% of *A. unedo* extract. Maximum force and negative area values presented in bars are expressed as mean ± standard deviation (*n * = 3). T0: 0 days; T60: 60 days. Different letters for the same parameter and formula indicate significant differences (*p* < 0.05).

Figure 4 presents the texture evaluation results of the formulations developed.

Figure 4A shows the F_max_ results (maximum force, corresponding to firmness) for the different formulas, while Figure 4B summarizes the results of adhesiveness. The firmness of formulations is influenced by the presence of the antioxidant extract, leading to a decrease in the parameter. The extract presence also affects adhesiveness, with formula Y presenting a lower negative area.

Concerning storage, no significant differences (*p* > 0.05) were verified for the formulas’ X parameters. On the contrary, formula Y’s firmness and adhesiveness were both influenced, showing an increase in both measurements. 

Pinto et al. [7] produced a hydrogel with *C. sativa* bur extract and observed a decrease in firmness and an increase in adhesiveness. In this study, the formula containing the lowest concentration of *C. sativa* bur extract, at T30 and 20 °C, presented a F_max_ of 0.429 N [7]. The results of the formula with *C. sativa* bur extract follow the same pattern of the present work, with firmness and adhesiveness increasing over time.

#### 3.3.5. Rheology

Rheology is an area of physics focused on understanding how materials flow and deform [73]. Rheological behavior encompasses two polar ends: (i) elastic, denoting a substance’s capacity to revert to its initial shape after the applied force is removed; and (ii) viscous (or plastic), characteristic of ideal Newtonian liquids that halt deformation upon cessation of the applied force. Most viscoelastic substances occupy a middle ground, wherein their microstructural systems govern both viscosity and elasticity in reaction to diverse flow conditions [74].

##### Viscosity

**Figure 5:**
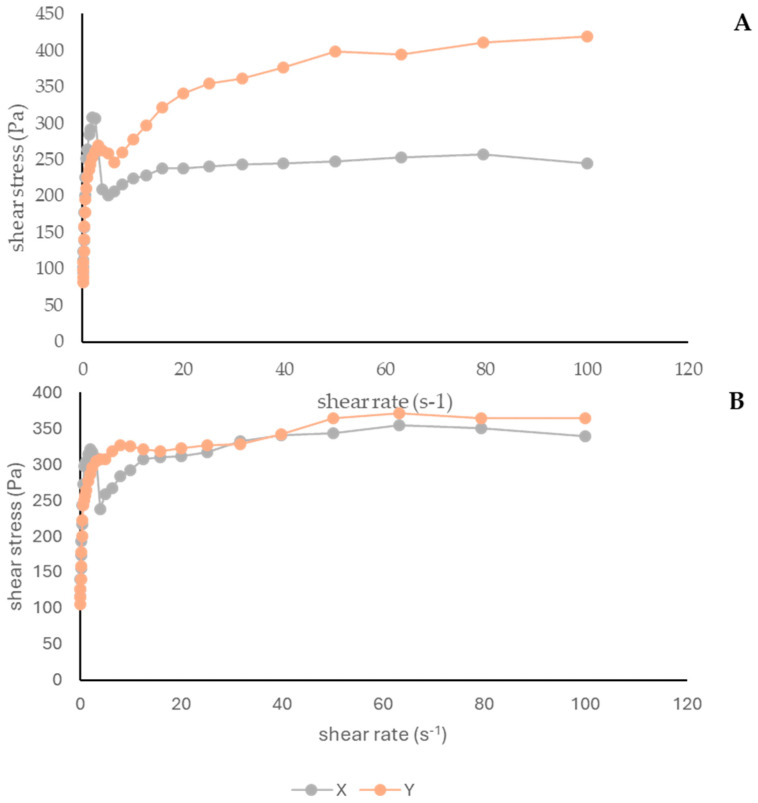
Rheograms of creams at 0 days (**A**) and 60 days (**B**). X: cream without the *A. unedo* extract; Y cream containing 10% of *A. unedo* extract.

Viscosity refers to a formulation’s resistance to deformation or flow, influenced by shear stress, as well as physicochemical properties and temperature. Viscosity evaluation is important not only to assess consumer satisfaction but also to estimate formulation’s stability [5]. Figure 5A,B represent the variation of shear stress versus shear rate on 0 and 60 days, respectively.

Both formulas displayed non-Newtonian pseudoplastic behavior, as shown in Figure 5, where viscosity decreases with an increase in shear rate. Pseudoplastic behavior is a desirable characteristic in cosmetic products because it facilitates the skin application and promotes the penetration of the product [5,75]. The shear stress of formulas X and Y were 245.1 and 419.3 Pa, respectively. This shows that the presence of the extract increases resistance to deformation.

Comparing the results of T0 and T60 for formula Y at higher shear rates, it is possible to attest that the formulation with extract showed a decrease in shear stress after 60 days of storage. Similar behavior has been observed in cosmetic formulations incorporating antioxidant extracts from plants. For example, the o/w semisolid formulation with *C. sativa* shell extract produced by Pinto et al. [5] was characterized by a pseudoplastic behavior. In another study, Pinto et al. [7] developed a hydrogel with *C. sativa* bur extract, and observed a similar behavior. Censi et al. [2] produced an o/w emulsion incorporating açai extract, also attesting to a pseudoplastic behavior. 

##### Thixotropy

Thixotropy corresponds to a decrease in viscosity over time when subjected to shear stress, followed by a gradual recovery once the stress is removed. A material that exhibits thixotropy undergoes a transition towards increased fluidity, while applied forces prolong it. Importantly, this transformation is reversible [74]. This is an important parameter in cosmetics since it facilitates spreading from the package, preventing run-off of the product [67].

**Figure 6:**
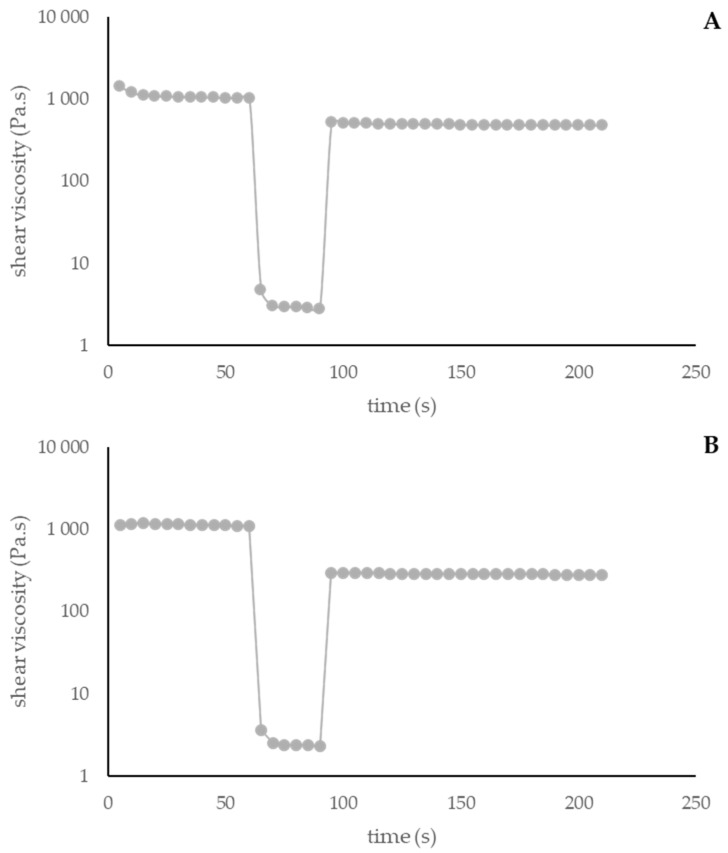
Thixotropy of Formula X at 0 days (**A**) and 60 days (**B**). X: cream without *A. unedo* extract.

**Figure 7:**
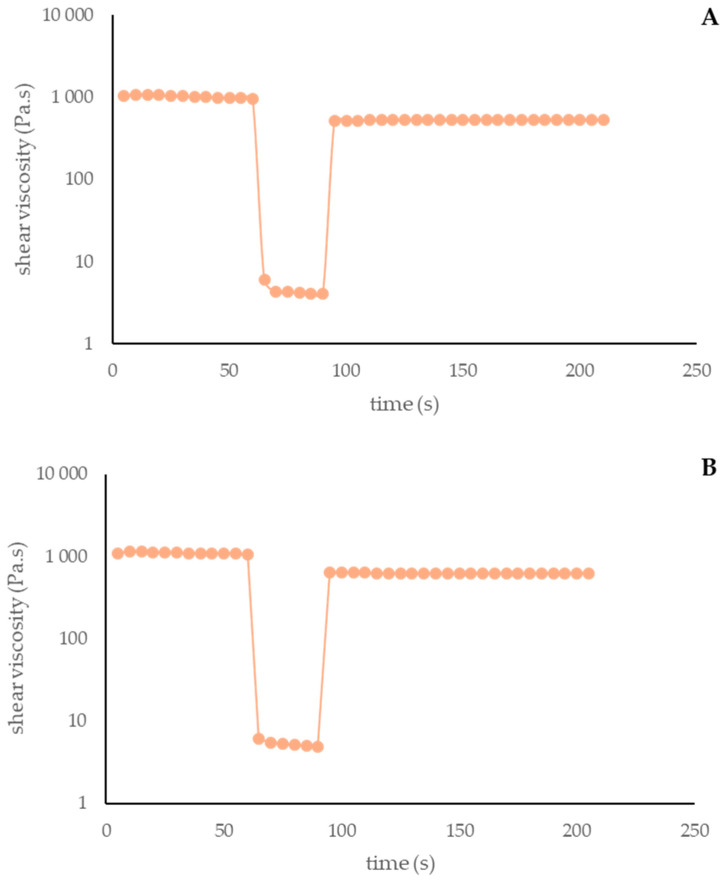
Thixotropy of Formula Y at 0 days (**A**) and 60 days (**B**). Y: cream containing 10% of *A. unedo* extract.

Figure 6 and Figure 7 represent the thixotropic behavior of both formulas at T0 and T60.

Both formulations evidenced thixotropic behavior, which was maintained over time. Comparing Formulas X and Y, it is possible to conclude that the extract reduced the formulation thixotropy after 60 days of storage.

##### Amplitude Sweep Test

**Figure 8:**
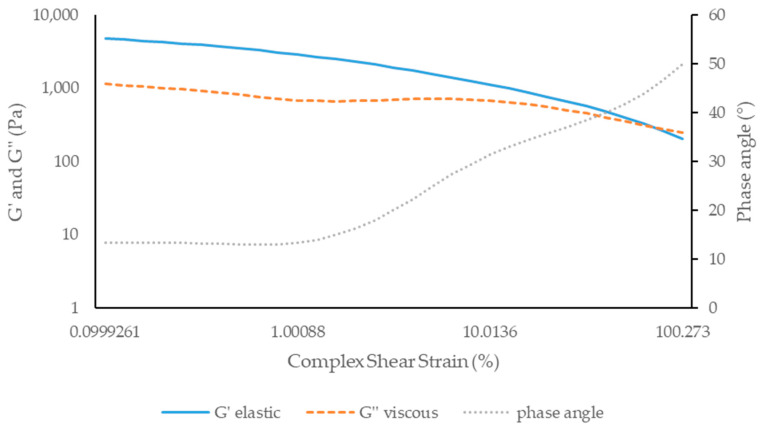
Amplitude sweep test of formula X: cream without the *A. unedo* extract.

**Figure 9:**
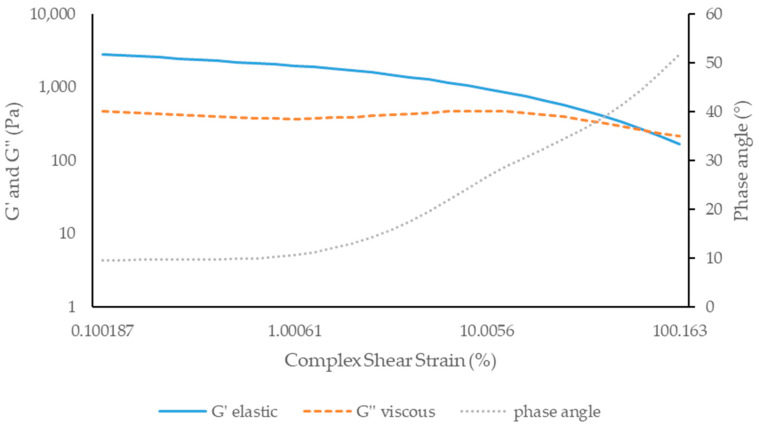
Amplitude sweep test of formula Y: cream containing 10% of *A. unedo* extract.

According to Figure 8 and Figure 9, the elastic component (G’) is predominant over the viscous component (G’’) for both formulations, with both formulations initially appearing as a solid viscoelastic or an apparent solid. For all formulations studied, the phase angle was between 0 and 90° (Formula X: 39.93° and Formula Y: 38.73°), reinforcing the viscoelastic character. Furthermore, it was found that, up to approximately 1% of deformation, no major changes occurred in the G’ and G’’ components of the creams.

## 4. Conclusions

This work developed a formulation composed by beeswax and *A. unedo* fruit extract obtained by UAE. Both ingredients were sourced from the Natural Park of Montesinho, a protected area. Beeswax presented a total of 14 fatty acids, mainly SFA, attesting its ideal composition to be used in cosmetic formulations, namely as an occlusive agent. The extract was optimized considering different times, and the 60-min extract proved to be the most effective in nearly all assays. Additionally, the cell viability assay performed in keratinocytes revealed the absence of toxicity in concentrations between 125 and 1000 µg/mL for this extract. Regarding the polyphenolic content, phenolic acids were predominant, followed by flavan-3-ols, tannins, flavonols, and anthocyanins. Moreover, no values exceeding the quantification limits were detected for the diverse set of contaminants assessed, confirming the *A. unedo* extracts’ safeness in the context of the studied groups of compounds. However, one OPE (TCP) was quantified in beeswax, even though these compounds are not currently included in the regulations. These findings highlight the need to update Regulation (EC) No 1223/2009 to address emerging pollutants and ensure the safety of cosmetic products and the protection of public health. The formulations were stored at room temperature and analyzed at time 0 and after 60 days of storage regarding different parameters. The accelerated stability tests showed that the formulations developed maintained their integrity. 

Through this work, rigorous safety control and assessment were conducted to ensure that the formulation met the stringent regulatory requirements without risks to consumers, supporting the ethical use of natural resources in cosmetic products. Most importantly, local matrices from a protected area were studied and valorized, considering green principles. Further work should be focused on studying the absence of skin irritation through 3D skin models, as well as through in vivo assays in volunteers to test claims and assess consumers’ acceptance.

## Figures and Tables

**Table 1:** Composition of the hydrophilic cream (%, *w*/*w*).

Ingredients	X	Y
Glycerin	10.0	10.0
Kathon^®^ CG	0.1	0.1
Tween^®^ 80	5.0	5.0
Carbopol^®^ 940	0.6	0.6
*A. unedo* extract	-	10.0
Beeswax	2.0	2.0
Cetyl Alcohol	15.0	15.0
Triethanolamine	q.s.	q.s.
Purified Water	q.s. 100.0	q.s. 100.0

q.s.: quantity sufficient; X: cream containing 10% of *A. unedo* extract; Y: cream without the *A. unedo* extract.

**Table 2:** Fatty acids (expressed as % of total FA) of beeswax samples. Values are expressed as mean ± standard deviation (*n* = 3).

	% Total of FA	RSD %
SFA		
C10:0	0.05 ± 1.79 × 10^−5^	4
C12:0	0.17 ± 4.78 × 10^−5^	3
C14:0	0.21 ± 2.38 × 10^−4^	11
C16:0	63.60 ± 1.13 × 10^−2^	2
C18:0	2.15 ± 1.99 × 10^−4^	1
C20:0	0.56 ± 6.11 × 10^−4^	11
C22:0	3.72 ± 7.33 × 10^−5^	0
C23:0	0.17 ± 2.04 × 10^−4^	12
C24:0	3.53 ± 2.06 × 10^−3^	6
MUFA		
C16:1 n-7	0.18 ± 2.79 × 10^−4^	16
C18:1 n-9c	15.55 ± 3.05 × 10^−3^	2
C20:1 n-9	0.42 ± 5.82 × 10^−4^	14
PUFA		
C18:2 n-6c	8.79 ± 8.03 × 10^−3^	9
C18:3 n-3	0.91 ± 7.00 × 10^−4^	8
Σ SFA	74.10 ± 1.01 × 10^−2^	1
Σ MUFA	16.15 ± 2.75 × 10^−3^	2
Σ PUFA	9.70 ± 7.33 × 10^−3^	8
Σ ω3	0.91 ± 7.00 × 10^−4^	8
Σ ω6	8.79 ± 8.03 × 10^−3^	9

SFA: saturated fatty acids; MUFA: monounsaturated fatty acids; PUFA: polyunsaturated fatty acids.

**Table 3:** Contaminants detected in beeswax through GC analysis and respective analytical parameters.

Chemical Class	Contaminant	Retention Time (min)	Concentration (µg/L)	R^2^	LOD (µg/L)	LOQ (µg/L)
OPEs	TToP = TCP	48.2	8.38	0.999	0.780	2.58

LOD: limit of detection.

**Table 4:** In vitro antioxidant/antiradical activity, evaluated by ABTS, DPPH, and FRAP assays, and TPC of *A. unedo* L. extracts obtained by UAE. Values are expressed as mean ± standard deviation (*n* = 3).

Extraction Time (min)	ABTS (mg AAE/g dw)	DPPH (mg TE/g dw)	FRAP (µmol FSE/g dw)	TPC (mg GAE/g dw)
15	26.78 ± 1.86 ^b^	37.62 ± 3.19 ^b^	353.69 ± 16.05 ^c^	24.69 ± 1.87 ^c^
30	29.44 ± 1.70 ^a^	43.58 ± 3.33 ^a^	393.76 ± 14.92 ^b^	29.00 ± 1.08 ^a,b^
45	28.10 ± 1.02 ^a,b^	38.83 ± 1.20 ^b^	363.55 ± 17.67 ^c^	26.02 ± 0.85 ^c^
60	30.36 ± 1.67 ^a^	43.83 ± 2.95 ^a^	415.61 ± 14.29 ^a^	30.27 ± 1.31 ^a^
90	29.91 ± 2.38 ^a^	40.02 ± 2.76 ^a,b^	365.06 ± 15.68 ^c^	28.05 ± 0.72 ^b^

Different letters in the same column indicate significant differences (*p* < 0.05).

**Table 5:** Reactive oxygen species scavenging capacity of the positive controls and *A. unedo* L. extracts obtained by UAE. Values are expressed as mean ± standard deviation (*n* = 3).

Extraction Time (min)	HOCl	O_2_^•−^	ROO^•^ (µmol TE/mg dw)
	IC_50_ (µg/mL)		
15	17.23 ± 2.33 ^b^	342.44 ± 30.41 ^e^	0.23 ± 0.01 ^c^
30	31.63 ± 2.59 ^d^	288.25 ± 17.43 ^d^	0.11 ± 0.02 ^c^
45	24.43 ± 2.38 ^c^	176.25 ± 21.92 ^c^	0.21 ± 0.03 ^c^
60	19.78 ± 2.24 ^b^	90.51 ± 16.43 ^b^	0.19 ± 0.05 ^c^
90	29.60 ± 0.34 ^d^	156.82 ± 5.96 ^c^	0.17 ± 0.03 ^c^
Positive control			
Gallic Acid	2.60 ± 0.14 ^a^	6.34 ± 0.53 ^a^	2.45 ± 0.54 ^b^
Catechin	0.20 ± 0.03 ^a^	18.01 ± 0.77 ^a^	6.37 ± 2.32 ^a^

Different letters in the same column indicate significant differences (*p* < 0.05).

**Table 6:** Identification and quantification of the phytochemical profile of the *A. unedo* L. extracts, obtained by UAE, through HPLC-PDA analysis. Values are expressed as mean ± standard deviation (*n * = 3).

Compound	15′	30′	45′	60′	90′
	mg/100 g				
Phenolic acids					
Gallic acid	1528 ± 76	2033 ± 102	2238 ± 112	2180 ± 109	2063 ± 103
Protocatechuic acid	ND	ND	ND	ND	4.52 ± 0.23
Neochlorogenic acid	1.87 ± 0.09	1.90 ± 0.09	2.92 ± 0.15	0.93 ± 0.05	2.46 ± 0.12
Caftaric acid	2.19 ± 0.11	1.35 ± 0.07	9.46 ± 0.47	2.36 ± 0.12	1.71 ± 0.09
Chlorogenic acid	15.73 ± 0.79	5.47 ± 0.27	8.02 ± 0.40	6.61 ± 0.33	6.34 ± 0.32
4-*O*-caffeoylquinic acid	12.38 ± 0.62	2.54 ± 0.13	9.12 ± 0.46	14.20 ± 0.71	11.94 ± 0.60
Vanillic acid	3.08 ± 0.15	2.36 ± 0.12	2.46 ± 0.12	2.46 ± 0.12	2.20 ± 0.11
Caffeic acid	0.80 ± 0.04	0.83 ± 0.04	1.31 ± 0.07	0.22 ± 0.01	ND
Syringic acid	6.79 ± 0.34	0.17 ± 0.01	3.58 ± 0.18	0.37 ± 0.02	ND
*p*-Coumaric acid	1.06 ± 0.05	0.65 ± 0.03	0.56 ± 0.03	0.62 ± 0.03	0.61 ± 0.03
Ferulic acid	13.85 ± 0.69	1.22 ± 0.06	7.68 ± 0.38	6.09 ± 0.30	4.22 ± 0.21
Sinapic acid	2.09 ± 0.10	2.00 ± 0.10	2.50 ± 0.13	2.77 ± 0.14	2.57 ± 0.13
3,5-di-caffeoylquinic acid	0.99 ± 0.05	ND	ND	ND	ND
Ellagic acid	7.34 ± 0.37	1.65 ± 0.08	3.29 ± 0.16	1.64 ± 0.08	1.64 ± 0.08
4,5-di-*O*-caffeoylquinic acid	2.09 ± 0.10	1.58 ± 0.08	2.63 ± 0.13	1.43 ± 0.07	1.54 ± 0.08
Σ Phenolic acids	1598	2055	2292	2220	2103
Flavonols					
Quercetin-3-*O*-galactoside	20.19 ± 1.01	8.71 ± 0.44	13.62 ± 0.68	13.96 ± 0.70	11.47 ± 0.57
Myricetin	3.71 ± 0.19	1.49 ± 0.07	2.22 ± 0.11	2.47 ± 0.12	2.36 ± 0.12
Σ Flavonols	23.90	10.20	15.84	16.43	13.83
Flavan-3-ols					
Catechin	36.78 ± 1.84	17.48 ± 0.87	16.72 ± 0.84	15.57 ± 0.78	14.94 ± 0.75
Epicatechin	37.23 ± 1.86	1.74 ± 0.09	20.12 ± 1.01	15.11 ± 0.76	9.80 ± 0.49
(-)-gallocatechin	24.0 ± 1.20	19.09 ± 0.95	20.76 ± 1.04	20.77 ± 1.04	21.5 ± 1.08
(-)-epigallocatechin	64.3 ± 3.22	17.92 ± 0.90	139 ± 6.93	147 ± 7.35	111 ± 5.57
(-)-epicatechin gallate	<LOQ	<LOQ	<LOD	<LOQ	<LOQ
	(-)-catechin gallate	<LOD	<LOQ	<LOQ	<LOQ
Σ Flavan-3-ols	162.31	56.23	196.60	198.45	157.24
Stilbenes					
trans-polydatin	1.16 ± 0.06	ND	ND	1.01 ± 0.05	0.59 ± 0.03
Resveratrol	0.99 ± 0.05	ND	ND	ND	ND
Σ Stilbenes	2.15	ND	ND	1.01	0.59
Anthocyanins					
Pelargonidin chloride 3	<LOQ	<LOQ	<LOQ	<LOD	<LOQ
Delphinidin-3-*O*-glucoside chloride	19.8 ± 0.99	<LOD	ND	<LOD	<LOD
Cyanidin-3-*O*-galactoside chloride	<LOQ	<LOD	ND	<LOD	<LOD
Cyanidin-3-*O*-glucoside chloride	6.98 ± 0.35	6.11 ± 0.31	7.22 ± 0.36	7.23 ± 0.36	7.69 ± 0.38
Malvidin-3,5-di-*O*-glucoside chloride	<LOQ	<LOD	<LOQ	<LOQ	<LOQ
Petunidin-3-*O*-glucoside chloride	ND	<LOQ	<LOQ	<LOD	<LOD
Peonidin-3-*O*-glucoside chloride	2.34 ± 0.12	ND	ND	<LOQ	<LOQ
Malvidin-3-*O*-glucoside chloride	<LOD	4.00 ± 0.20	<LOD	<LOD	<LOD
Σ Anthocyanins	29.12	10.11	7.22	7.23	7.69
Tannins					
Procyanidin B1	10.7 ± 0.53	6.69 ± 0.33	7.54 ± 0.38	7.69 ± 0.38	7.70 ± 0.39
Procyanidin B2	49.4 ± 2.47	48.65 ± 2.43	48.77 ± 2.44	46.63 ± 2.33	49.81 ± 2.49
Σ Tannins	60.10	55.34	56.31	54.32	49.81
Other					
Caffeine	8.08 ± 0.40	3.80 ± 0.19	3.85 ± 0.19	3.83 ± 0.19	3.73 ± 0.19
Phloridzin	6.68 ± 0.33	0.35 ± 0.02	2.68 ± 0.13	0.54 ± 0.03	0.78 ± 0.04
Naringin	0.78 ± 0.04	ND	ND	ND	ND
Σ Other	15.54	4.15	6.53	4.37	4.51
Σ Total	1891	2191	2574	2502	2336

15′, 30′, 45′, 60′ and 90′: extraction time (min); LOD: limit of detection; LOQ: limit of quantification; ND: not determined.

**Table 7:** Effects of *A. unedo* L. extracts, obtained by UAE, and exposure on the viability of HaCaT cell line at different concentrations, as measured by the MTT assay. Values are expressed as mean ± standard deviation (*n * = 4).

Extraction Time (min)	Concentrations (µg/mL)			
	125	250	500	1000
15	97.97 ± 19.23 ^a^	92.15 ± 16.22 ^a^	86.69 ± 18.74 ^a^	88.58 ± 14.55 ^a^
30	80.36 ± 2.37 ^a^	84.70 ± 13.51 ^a^	53.20 ± 11.04 ^b^	57.24 ± 6.16 ^b^
45	48.17 ± 8.58 ^a^	27.06 ± 4.12 ^b^	24.85 ± 2.48 ^b^	31.24 ± 5.07 ^b^
60	105.36 ± 8.27 ^a^	99.16 ± 6.63 ^a^	93.34 ± 4.99 ^a^	90.17 ± 14.90 ^a^
90	89.78 ± 4.54 ^a^	85.26 ± 9.17 ^a^	78.93 ± 3.93 ^a,b^	62.67 ± 8.42 ^b^
Negative control	0.00 ± 0.46			
Positive control	103.68 ± 16.57			

Different letters in the same column indicate significant differences (*p* < 0.05).

**Table 8:** Contaminants detected in *A. unedo* berries’ 15-min extract through GC analysis and analytical parameters.

Chemical Class	Contaminant	Retention Time (min)	Concentration (µg/L)	R^2^	LOD (µg/L)	LOQ (µg/L)
OCPs	p,p’-DDE	31.6	<LOD	0.999	0.709	2.37

LOD: limit of detection.

**Table 9:** Color (L*, a*, b*, and C*) and pH variation of formulation with (Y) and without (X) *A. unedo* extract over 60 days. Values are expressed as mean ± standard deviation (*n * = 3).

		Color			pH
	C*	L* (Lightness)	a* (Redness)	b* (Yellowness)	
X					
T0	7.81	84.10 ^b^	−2.42 ^b^	7.43 ^a^	6.61 ^a^
T60	9.24	82.68 ^a^	−2.30 ^a^	8.96 ^b^	6.68 ^a^
Y					
T0	11.41	82.96 ^b^	−2.67 ^b^	11.09 ^a^	6.59 ^a^
T60	11.30	79.20 ^a^	−1.58 ^a^	11.19 ^b^	6.63 ^a^

T0: 0 days; T60: 60 days. Different letters in the same column indicate significant differences (*p* < 0.05).

## Data Availability

Data are contained within the article.
